# Pheromone gland development and monoterpenoid synthesis specific to oviparous females in the pea aphid

**DOI:** 10.1186/s40851-018-0092-0

**Published:** 2018-05-11

**Authors:** Koki Murano, Kota Ogawa, Tomonari Kaji, Toru Miura

**Affiliations:** 1 Laboratory of Ecological Genetics, Graduate School of Environmental Science, Hokkaido University, Sapporo, Hokkaido 060-0810 Japan; 2 Laboratory of Functional Genomics, National Institute for Basic Biology, Okazaki, Aichi 444-8585 Japan; 3 Bamfield Marine Science Centre, 100 Pachena Rd, Bamfield, British Columbia V0R 1B0 Canada; 4 Misaki Marine Biological Station, School of Science, The University of Tokyo, 1024 Koajiro, Misaki, Miura, Kanagawa 238-0225 Japan

**Keywords:** Epidermal gland, Hind tibia, Mevalonate pathway, Oviparous female, Reproductive polyphenism, Parthenogenesis, Pea aphid, Scent plaque, Sex pheromone

## Abstract

**Background:**

Aphids display “cyclic parthenogenesis,” in which parthenogenetically and sexually reproducing morphs seasonally alternate in the aphid annual life cycles. There are various characteristics that differ between asexual viviparous and sexual oviparous females. In oviparous females, swollen cuticular structures (~ 10 μm in diameter), called “scent plaques,” are scattered on the surface of hind tibias, and secrete monoterpenoid sex pheromones. However, the developmental processes of the pheromone glands and the biosynthetic pathways of monoterpenoid pheromones have yet to be elucidated.

**Results:**

Comparisons of the developmental processes that form hind tibias between sexual and parthenogenetic females revealed that, in sexual females, the epithelial tissues in proximal parts of hind tibias become columnar in fourth instar nymphs, and circular pheromone glands with Class 1 gland cells appear in adults, although they do not appear in parthenogenetic females. Furthermore, by comparing the expression levels of genes involved in the mevalonate pathway, which is required for monoterpenoid synthesis, we show that genes that encode the downstream enzymes in the pathway are highly expressed in hind tibias of sexual females.

**Conclusion:**

Glandular tissues of scent plaque are differentiated from the fourth instar in sexual females, while parthenogenetic females lack the glandular cells. Only the downstream steps of the mevalonate pathway appear to occur in scent plaques on hind tibias of sexual females, although the upstream steps may occur somewhere in other body parts.

**Electronic supplementary material:**

The online version of this article (10.1186/s40851-018-0092-0) contains supplementary material, which is available to authorized users.

## Background

**Fig. 1:**
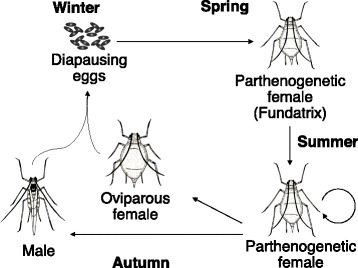
Cyclic parthenogenesis in the pea aphid. Oviparous sexual females appear only once per year, while parthenogenetic females occur throughout the rest of the annual life cycle

Most aphid species show cyclic parthenogenesis, in which both sexual and asexual reproduction are performed depending on season [1–4] (Fig. 1). From spring to summer, aphids proliferate dramatically by viviparous parthenogenesis. In autumn, parthenogenetic females produce oviparous females and males in response to short photoperiods [5]. Oviparous females copulate with males and then lay diapausing eggs. The following spring, parthenogenetic females known as “fundatrices” or “stem mothers” hatch from the diapausing eggs. Cyclical parthenogenesis is a case of reproductive polyphenism; both asexual and sexual females occur from the same genetic background in response to different environmental conditions [6–8]. In aphids, sexual and asexual females have different characteristics that are adaptive to each respective reproductive mode and appear through two different developmental processes. Although sexual females cannot reproduce without mating with males, parthenogenetic (asexual) females can produce clonal offspring without males. It has thus been suggested that characteristics for mating behaviors, such as attraction of males, are unnecessary for parthenogenetic females.

Sexual aphid females are known to secrete sex pheromones to attract males [9–12]. In many aphid species, including the pea aphid *Acyrthosiphon pisum*, two monoterpenoids, (4aS, 7S, 7aR)-nepetalactone and (1R, 4aS, 7S, 7aR)-nepetalactol, have been identified as sex pheromones [13–17]. The sex pheromones are thought to be secreted from circular bulge structures, called “scent plaques,” scattered on their hind tibias, which was supported by the findings that oviparous females whose hind legs were mutilated attracted fewer males [9, 10, 18, 19]. Since scent plaques are the sex pheromone glands of aphids, the epithelial cells under the scent plaques should be differentiated into specific gland cells for secreting sex pheromones, although the detailed histological structures of gland cells and the developmental process remain unknown.

Generally, terpenoids can be synthesized by several biosynthetic pathways. The mevalonate pathway is the most well-known, and is essential to isoprenoid synthesis in eukaryotes, in which acetyl-CoA is converted to isopentenyl diphosphate (IPP) and dimethylallyl diphosphate (DMAPP) through several enzymatic reactions [20]. In some insects, monoterpenoid pheromones have been shown to be synthesized through the mevalonate pathway [21, 22]. We hypothesized that the mevalonate pathway is also utilized for sex pheromone synthesis in aphids.

In the present study, hind-tibia structure and development were compared between oviparous and parthenogenetic females, and detailed scent plaque structures were histologically observed to examine whether the scent plaque possesses characteristics of a pheromone gland. In addition, although the place of secretion is thought to be scent plaques, the places of reactions in the mevalonate pathway may not necessarily be scent plaques. In order to examine from which step of the mevalonate pathway was localized in hind tibias of oviparous aphids, expression levels of genes that encode enzymes in the mevalonate pathway were compared by realtime quantitative PCR (qPCR). Based on the KEGG pathway database (http://www.genome.jp/kegg/pathway.html), seven major enzymes were chosen: acetoacetyl-CoA thiolase (AACT), HMG-CoA (3-hydroxy-3-methylglutaryl-coenzyme A) synthetase (HMGS), HMG-CoA reductase (HMGR), mevalonate kinase (MevK), phosphomevalonate kinase (MevPK), mevalonate 5-diphosphate decarboxylase (MevPPD), and isopentenyl pyrophosphate isomerase (IPPI) [23–25]. In addition, farnesyl diphosphate synthetase (FPPS) was also included, as it converts the final products in the mevalonate pathway, i.e., IPP and DMAPP, into geranyl diphosphate (GPP), which is a monoterpenoid precursor, as shown in the bark beetle *Ips pini* [22, 24, 26].

## Methods

### Insects

The ApL strain of the pea aphid, *Acrthosiphon pisum*, which was suitable to the induction of sexual generation, was used in this study. This strain was originally obtained from Sapporo, Hokkaido (referred to as Sap05Ms2 in [27]) and used as the ApL strain in the following studies [28]. The *A. pisum* genome was sequenced and is publicly available [29]. During parthenogenetic generations, aphids were reared in plastic tubes (diameter: 2.5 cm, height: 10 cm), in each of which a vetch seedling (*Vicia faba*) was placed on wet vermiculite. The tubes were kept under long-day and room-temperature conditions (16 h light: 8 h dark; 20 °C) [30].

### Induction of parthenogenetic and oviparous females

The induction of viviparous and oviparous females in the ApL strain was performed following the protocols previously described [28]. Briefly, a first-instar aphid produced by a single viviparous parthenogenetic female was isolated and reared in a rectangular plastic case (width: 6 cm, height: 10 cm, depth: 2.5 cm) with a vetch seedling at 15 °C under short-day conditions (8 h light: 16 h dark). After becoming an adult, the parthenogenetic female produced approximately 30 first-instar nymphs, defined as Short-day Generation 1 (“SG1”), during the first 16 days after the onset of larviposition [28]. The SG1 parthenogenetic females were again reared under short-day and low-temperature conditions (8 h light: 16 h dark; 15 °C) until larviposition. SG1 females produced exclusively sexual oviparous females during the first six days after the onset of larviposition, which were collected and reared in rectangular cases (100 mm × 65 mm × 28 mm) under the same day-length conditions (8 h light: 16 h dark; 15 °C).

### Scanning electron microscopy (SEM)

To examine the detailed morphological features of hind legs in third, fourth, and fifth (adult) instars of female aphids, the surface structures were observed by SEM. Specimens for SEM observations were prepared as previously described [31]. Briefly, aphid samples were fixed using a microwave oven, transferred into increasing concentrations of ethanol, and then transferred into hexamethyldisilazane. Subsequently, samples were transferred into t-butanol and freeze-dried using a freeze dryer (ES-2030; Hitachi, Tokyo, Japan), and legs were dissected from the bodies. Then, the legs were coated with gold ion using an ion sputter (E-1010; Hitachi, Tokyo, Japan). Detailed morphological characteristics of legs were then observed by SEM (JSM-5510LV; JEOL, Tokyo, Japan).

### Paraffin sectioning

To determine inner morphological features of hind legs, female legs of third, fourth, and fifth instars were observed using paraffin sections. As described previously [31], aphid legs fixed in FAA fixative (formalin-acetic acid-alcohol fixative: formalin: acetic acid: ethanol = 6:1:16) were dehydrated in increasing concentrations of ethanol and embedded in paraplast (Sigma-Aldrich, St. Louis, MO, USA). Sections (5-μm thick) were processed using a microtome. Sections were stained with hematoxylin and eosin. Tissues were observed under a light microscope (BZ-9000E; Keyence, Osaka, Japan).

### Transmission electron microscopy (TEM)

To determine inner ultrastructure of epidermal tissue from hind tibias of fourth and fifth instars of female aphids, TEM observations were performed. Specimens were prepared following the protocols described in [32]. Aphid hind tibias were initially fixed in 2% glutaraldehyde and 2% paraformaldehyde in 0.2 M cacodylate sodium buffer (pH 7.4) at 4 °C for 5 h, postfixed in 2% osmium tetroxide in the same buffer for 2 h at 4 °C, and then dehydrated in increasing concentrations of acetone. Subsequently, the hind tibias were embedded in EPON 812 resin and polymerized. Ultra-thin sections were made using an ultramicrotome EM UC7 (Leica, Tokyo, Japan) with a diamond knife cryo 35-(wet) (DiATOME, Biel, Switzerland). The sections were then stained with 1% potassium permanganate solution for 2 min and 2% lead citrate for 3 min. The prepared sections were then observed by TEM (H-7500; Hitachi, Tokyo, Japan).

### Identification of the genes in the *A. pisum* genome

To compare expression levels of genes that encode enzymes involved in monoterpenoid synthesis, AACT, HMGS, HMGR, MevK, MevPK, MevPPD, IPPI, and FPPS were searched for in the pea aphid genome (Refseq from version_2.0 of the *A. pisum* genome; http://www.ncbi.nlm.nih.gov/) using protein sequences of orthologous genes in *Drosophila melanogaster* or *Tribolium castaneum* as queries for Blast search. To confirm the putative orthologous genes in aphids, neighbor-joining trees of protein sequences were constructed using MEGA7 [33] (http://www.megasoftware.net). To estimate node confidence, 1000 bootstrap replicates were performed. The confirmed enzyme genes and their gene IDs are listed in Table 1.

**Table 1:** List of enzyme genes involved in the monoterpenoid synthesis

Enzyme	Gene	NCBI-Gene ID	Reference nucleotide sequence	Reference protein sequence
Acetoacetyl-CoA thiolase	*AACT1*	100,161,259	XM_001951826.3	XP_001951861.2
	*AACT2*	100,161,636	XM_001944000.3	XP_001944035.2
	*AACT3*	100,164,451	XM_008182335.1	XP_008180557.1
	*AACT4*	100,165,942	XM_008186356.1	XP_008184578.1
	*AACT5*	100,162,815	XM_001945208.3	XP_001945243.1
HMG-CoA synthetase	*HMGS1*	100,161,670	XM.001945901.3	XP_001945936.1
	*HMGS2*	100,165,154	XM_008187497.1	XP_008185719.1
HMG-CoA reductase	*HMGR*	100,165,462	XM_001951859.3	XP_001951894.2
Mevalonate kinase	*MevK*	100,163,305	XM_008189330.1	XP_008187552.1
Phosphomevalonate kinase	*MevPK*	100,163,413	NM_001162102.2	NP_001155574.1
Mevalonate 5-diphosphate decarboxylase	*MevPPD1*	100,158,798	XM_008182114.1	XP_008180336.1
	*MevPPD2*	100,160,652	XM_001950387.3	XP_001950422.2
IPP isomerase	*IPPI1*	100,166,744	XM_008186747.1	XP_008184969.1
	*IPPI2*	100,167,596	XM_008181419.1	XP_008179641.1
Farnesyl diphospate synthetase	*FPPS*	100,144,905	NM 001126161.3	NP 001119633.3

### Realtime qRT-PCR

For real-time quantitative RT-PCR, total RNA was extracted by ISOGEN (Nippon Gene, Tokyo, Japan) with recombinant DNase1 (RNase-free) (TaKaRa, Shiga, Japan) from more than 40 hind tibias (20 aphid individuals) and three whole bodies of adult aphids. For biological replication, three replicated samples were prepared for each of the following four categories: (1) hind tibias of adult oviparous females, (2) hind tibias of adult parthenogenetic females, (3) whole bodies of adult oviparous females, and (4) whole bodies of adult parthenogenetic females. For each sample, extracted total RNA was reverse-transcribed with the High Capacity cDNA Reverse Transcription Kit based on the manufacturer’s instructions (Applied Biosystems, Foster City, CA, USA).

Primers for the target enzyme genes were designed using Primer Express software v3.0.1 (Applied Biosystems) (Additional file 1: Table S1). For *AACT1* and *AACT2*, the primers were designed to cover the conserved region shared by the two paralogs, because the DNA sequences of the two genes are too similar to design primers specific to each paralog.

Quantitative RT-PCR (qRT-PCR) was performed using a SYBR Green I Chemistry System and Sequence Detection System ABI PRISM 7500 (Applied Biosystems, Foster City, CA, USA). To determine the endogenous control of constitutive expression, the suitability of different putative reference genes was evaluated among 4 candidate reference genes, *glyceraldehyde-3-phosphate dehydrogenase* (*GAPDH*), *beta-actin, elongation factor 1 alpha* (*EF1a*), and *ribosomal protein L32* (*rpL32*), by three statistical software programs, geNorm [34], BestKeeper [35], and Normfinder [36]. As the results, the expression level of *GAPDH* was shown to be the most stable so that it was used as the reference gene.

Data acquisition and analyses were performed by ABI Prism 7500 v2.0.1 (Applied Biosystems, Foster City, CA, USA). Baselines and Ct values (threshold cycle) were set automatically. The relative standard curve method was used for qRT-PCR quantification, as described in User Bulletin 2 for the ABI Prism 7700 Sequence Detection System (Applied Biosystems, Foster City, CA, USA). To evaluate the significance of expression differences among the categories, Student’s t-tests (*p* < 0.01) were performed.

## Results

### Scanning electron microscopy on the hind tibia surface

**Fig. 2:**
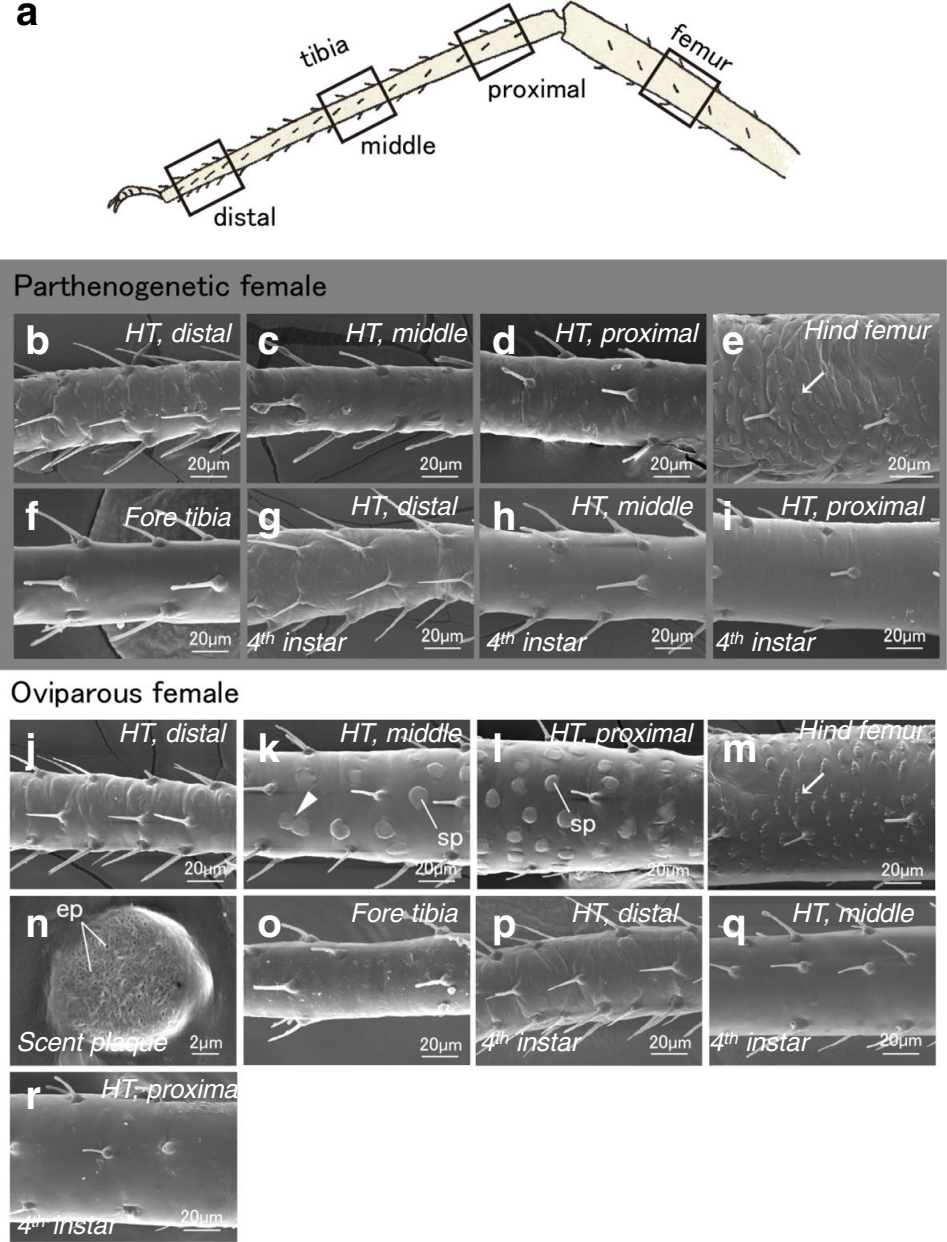
Comparisons of leg surface structures between parthenogenetic and oviparous females. **a**: Outline of observed parts. **b–d**: Hind tibia of an adult parthenogenetic female (distal, middle, and proximal parts). **e**: Hind femur of an adult parthenogenetic female. **f**: Foreleg tibia of an adult parthenogenetic female. **g–i**: Hind tibia of a fourth-instar parthenogenetic female (distal, middle, and proximal parts). **j–l**: Hind tibia of an adult oviparous female (distal, middle, and proximal parts). **m**: Hind femur of an adult oviparous female. **n**: Magnification (× 10) of a scent plaque. **o**: Foreleg tibia of an adult oviparous female. **p–r**: Hind tibia of a fourth-instar oviparous female. Arrows indicate scale-like structures, and arrowheads indicate scent plaques showing an “∞” shape. *sp.:* scent plaque, *ep:* epicuticular pore

First, hind legs of adult female aphids were observed and compared between two reproductive morphs (Fig. 2). In parthenogenetic females, the hind tibia surfaces had smooth features, and we did not observe any distinctive structures except for seta (Fig. 2b–d), and hind femurs possessed scale-like structures on the surface (Fig. 2e).

By contrast, in oviparous females, numerous circular bulge structures called scent plaques were scattered on the hind tibia surface, especially in proximal and medial parts (Fig. 2j–l). Most scent plaques ranged from 7 to 10 μm in diameter. Most occurred separate from one another, although some were attached, forming “∞” shapes (Fig. 2k). On each scent plaque, many small pores, called epicuticular pores, were observed (Fig. 2n). The surface structures on hind femurs were similar to those on parthenogenetic females, where scent plaques were absent (Fig. 2m). In addition, scent plaques were absent on the surfaces of fore and mid tibias, which had smooth surfaces, as seen in parthenogenetic females (Fig. 2f and o).

Furthermore, hind tibias were also observed in nymphs. In the fourth instar (last nymphal instar), no structural differences were found between the two morphs; i.e., scent plaques were not found on any leg part in either morph (Fig. 2g–i, p–r). The tibia surfaces were smooth, which was also observed in parthenogenetic adult females. Also, scent plaques were not found on hind tibias in any other nymphal instars (data not shown).

### Histological structures of hind tibial epidermis

**Fig. 3:**
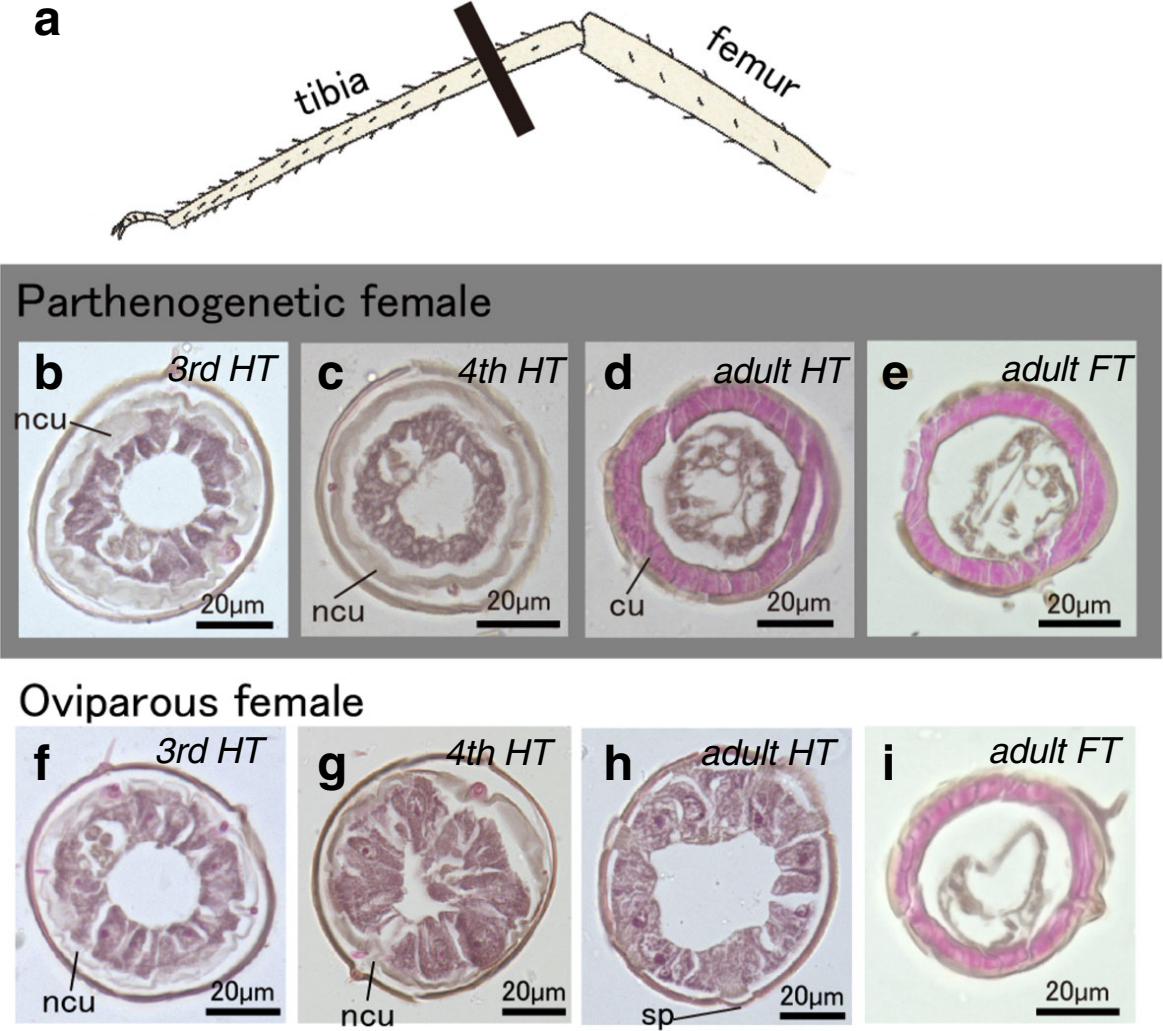
Inner structures of tibias in parthenogenetic and oviparous females. **a**: Diagram showing the histologically observed plane, which is indicated by a black bar. **b–d**: hind tibias (HT) of parthenogenetic females [third, fourth, and fifth (adult) instars]. **e**: Foreleg tibia (FT) in an adult parthenogenetic female. **f–h**: HT of oviparous females (third, fourth, and fifth instars). **i**: FT of an adult oviparous female. *cu:* cuticle, *ncu:* newly synthesized cuticle, *sp.:* scent plaque

The internal histological structures of hind tibias were first compared among adult individuals, and especially focused on the proximal part of hind tibias (Fig. 3). In parthenogenetic females, epithelial cells were flattened and showed sponge-like features (Fig. 3d). However, the cuticular layer was notably thickened (Fig. 3d). In oviparous females, in contrast, the epithelial cell shape was columnar (Fig. 3h). The outer surfaces of epithelial cells formed acylindrical projections, and connected to circular bulge structures, i.e., scent plaques (Fig. 3h). However, the hind tibial cuticle was thinner than that in parthenogenetic females (Additional file 2: Figure S1). In the case of fore-leg tibias of adult oviparous females, as seen in the hind tibias of parthenogenetic females, flattened epithelial cells and thick cuticles were observed in both morphs (Fig. 3e and i).

To understand the associated developmental processes, nymphal hind tibias were also observed. In the third-instar nymphs of both morphs, epithelial cell shape in hind tibias was columnar, and a newly formed cuticle was observed between the outer cuticle and epithelial cell layer (Fig. 3b and f). The structural differences between oviparous and parthenogenetic females were first observed in fourth-instar nymphs. In future oviparous females, the epithelial cell layers were more developed than those of asexual females (Fig. 3c and g).

### Epidermal cell ultrastructure

**Fig. 4:**
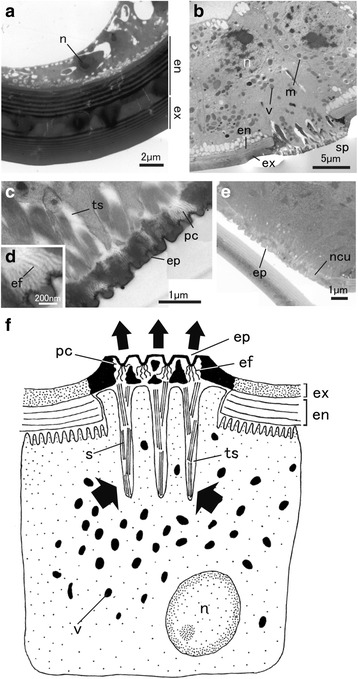
Ultrastructure of hind tibia epithelial tissues. **a**: Epidermis and cuticle of a hind tibia from an adult parthenogenetic female. **b–d**: Epidermis and cuticle of a hind tibia from an adult oviparous female (**b**: epithelial cells under the tibial cuticle, **c**: magnification (× 5) of a scent plaque, **d**: further magnification (× 10) of a pore canal). **e**: Hind tibia of a fourth-instar oviparous female. **f**: Diagram of detailed structures in a scent plaque of an adult oviparous female. Arrows in **f** indicate pheromone secretion. *n:* nuclei, *v:* secretory vesicle, *m:* cell membrane, *en:* endocuticle, *ex:* exocuticle, *sp:* scent plaque, *ts:* invaginated tubular structure, *ep:* epicuticular pore, *pc:* pore canal, *ef:* epicuticular filament, *ncu:* new cuticle

Ultrastructural observation by transmission electron microscope (TEM) revealed that, in adult parthenogenetic females, each epithelial cell in the hind tibia possessed a small nucleus and numerous vacuoles (Fig. 4a). These epithelial cells were flattened, and the boundary between cells was unclear, as the plasma membrane was not visible. In adult oviparous females, in contrast, many specific structures were found in the epithelial layer and cuticle of hind tibias. Each columnar epithelial cell contained a large nucleus and many secretory vesicles (Fig. 4b). The boundary between cells was clear, because the plasma membrane was clearly visible (Fig. 4b). Part of each epidermal cell penetrated the endocuticle and was connected to the exocuticle, forming a scent plaque (Fig. 4b). Under the circular bulge structure, the epithelial cells were deeply invaginated and had tubular structures. These tubular structures were filled with fibrous secretory materials (Fig. 4c). In the exocuticle, enlarged pore canals were observed (Fig. 4c). In the pore canals, many epicuticular filaments were observed (Fig. 4d).

In the fourth-instar nymphs of oviparous females, the epithelial tissues of hind tibias did not possess secretory vesicles, although epicuticular pores were already formed on newly formed cuticle (Fig. 4e). In parthenogenetic female nymphs, no epicuticular pores were observed on newly formed cuticle (data not shown).

### Terpenoid-synthesis genes

**Fig. 5:**
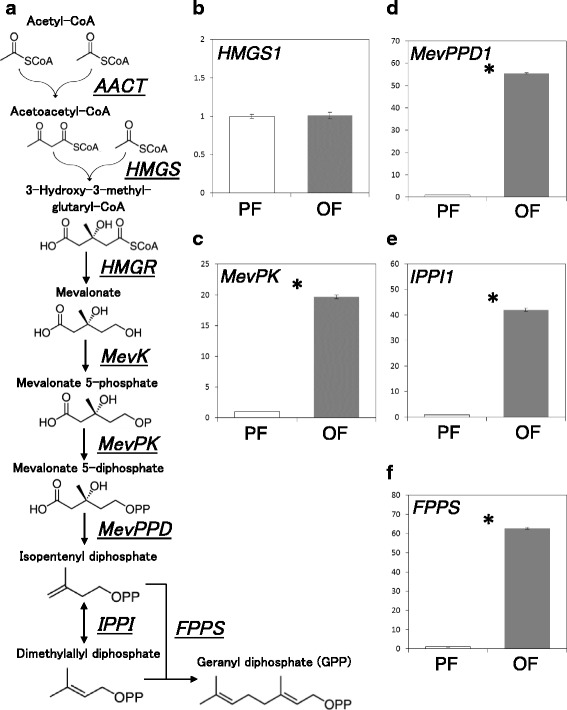
Comparisons of relative expression levels of enzyme genes in hind tibias. **a:** The mevalonate pathway with responsible catalytic enzymes. **b–f**: Expression levels of genes coding the mevalonate pathway enzymes. **b**: *HMG-CoA synthetase* (3-hydroxy-3-methylglutaryl-coenzyme A synthase, *HMGS1*), **c**: *phosphomevalonate kinase* (*MevPK*), **d**: *mevalonate 5-diphosphate decarboxylase* (*MevPPD1*), **e**: *isopentenyl pyrophosphate isomelase* (*IPPI1*), **f**: *farnesyl diphosphate synthetase* (*FPPS*). Blank and gray bars indicate parthenogenetic (PF) and oviparous females (OF), respectively. Vertical axes show relative expression levels. Asterisks indicate significant difference between parthenogenetic and oviparous females (Student’s t-test, *P* < 0.01)

The pea aphid orthologs of enzyme genes in the mevalonate pathway (*AACT, HMGS, HMGR, MevK, MevPPD, IPPI*, and *FPPS*) were confirmed from the aphid gene database **(**Table 1). *AACT* contained five paralogs, and *HMGS, MevPPD,* and *IPPI* contained two paralogs (Additional file 2: Figure S2). Realtime qRT-PCR analyses to compare expression levels of these genes between the two morphs in hind tibias and whole bodies of adult individuals revealed that in hind tibias, although *HMGS1* expression showed no significant difference between the two morphs (Student’s t-test, *P* < 0.01), *MevPK*, *MevPPD1*, *IPPI1*, and *FPPS* showed dramatically higher expression levels in oviparous females (Student’s t-test, *P* < 0.01) (Fig. 5). The expressional levels of these genes were 20–60 times higher in oviparous females. Expression levels of the other genes described were not detected.

In whole bodies, *AACT1&2, HMGS1,* and *MevPPD* did not show significant expression differences, but *MevPK, IPPI1, and FPPS* showed significantly higher expression levels in oviparous females (approximately two times higher; Student’s t-test, *P* < 0.01) (Additional file 2: Figure S3). The expression levels of the other genes (*AACT3–5, HMGS2, HMGR, MevK, MevPPD2,* and *IPPI2*) were not detected, probably due to very low levels of expression.

## Discussion

The present study describes the internal structures of the scent plaques associated with epidermal gland cells in the pea aphid. Epidermal gland cells are generally classified into three types, as follows: (1) Class 1 epidermal gland cells are monocellular and contact the glandular cuticle; (2) Class 2 epidermal gland cells do not contact the glandular cuticle, and transport secretory materials into adjacent Class 1 cells; and (3) Class 3 epidermal gland cells are multicellular, and consist of secretory cells and duct cells [37, 38]. In Class 1 epidermal glands, the glandular cuticle is often thin to facilitate transport of secretory molecules [37, 39]. In addition, enlarged pore canals, epicuticular filaments, and epicuticular pores are often formed in the glandular cuticle of Class 1 cells to facilitate diffusion of large molecules or retain secretory materials [37, 38, 40–42]. Based on these criteria, scent plaques in the pea aphid are suggested to be composed of glandular cuticle, and the epithelial cells under the scent plaques are classified as Class 1 gland cells, suggesting that sex pheromones are synthesized in the gland cells under the scent plaques. Thus, in the hind tibias of adult oviparous females, the sex pheromones are first transported and contained in invaginated tubular structures, and are secreted through the glandular cuticle (i.e., scent plaques) (Fig. 4f).

In general, the epidermis of adult insects is flattened, as adult insects generally do not synthesize new cuticles [38, 43]. Hind, fore, and mid tibias of adult parthenogenetic females have general adult features but the hind tibias lack scent plaques, which indicates that the epithelial cells in parthenogenetic females are incapable of secreting sex pheromones.

Monoterpene biosynthesis is essential for plants to synthesize defense oil and resin [26]. In animals, however, monoterpene biosynthesis was thought to be rare [21]. In contrast, recent studies revealed that several beetles (*I. pini*, *Dendroctonus jeffreyi*, *Phaedon cochleariae,* and *Gastrophysa viridula*) used the mevalonate pathway to synthesize monoterpenes [21, 44–49]. In addition, the FPPS protein in the peach-potato aphids, *Myzus persicae*, was reported to convert the final products of mevalonate pathway to GPP [50, 51]. Therefore, the qRT-PCR results (Fig. 5) indicated that the downstream enzymes (MevPK, MevPPD, and IPPI) and FPPS were responsible for monoterpene biosynthesis in oviparous females **(**Figs. 2–4**)**, which is consistent with the fact that only oviparous females possess Class 1 gland cells on hind tibias. These results indicate that oviparous females synthesize monoterpenoid sex pheromones by up-regulating gene expression of downstream enzyme genes in the Class 1 epidermal gland cells of hind tibias. In contrast, the expression levels of all paralogous genes of *AACT, HMGR*, and *MevK* were not detected from hind tibias. However, the expression of AACT were detected in whole bodies of both morphs (Additional file 2: Figure S3). Based on these results, we suggest that the upstream substrates are also synthesized through the mevalonate pathway somewhere in the body, and the products (precursors of terpenoids) are transported to the gland cells of hind tibias to facilitate the downstream cascades of terpenoid synthesis.

As for gene expression in whole bodies, the expression levels of mevalonate-pathway genes were relatively high even in parthenogenetic females. This suggests that the pathway is also important for parthenogenetic females. For example, it is known that the mevalonate pathway is also crucial for juvenile hormone (JH) biosynthesis in insects, which suggests that expression levels in whole bodies may be caused by JH biosynthesis in the corpora allata. Juvenile hormone is reported to play important roles in the production of parthenogenetic females [52], and some JH-related genes are down-regulated under short-day length, when sexual females are produced [53]. Although these JH actions are only known to affect the next generations, JH should also be required for the postembryonic development (i.e., moultings) both in the parthenogenetic and sexual females, so that expressions of the mevalonate-pathway genes were detected in whole bodies.

Parthenogenesis is thought to have evolved in aphid life cycles about 200 million years ago [2, 54–57]. In addition, scent plaques are found in the most Aphididae species [9, 10, 58], and it was reported that two monoterpenoid components are used as sex pheromones in many aphid species [19, 59]. These suggest that hind tibias with scent plaques is an ancestral characteristic in Aphididae. Therefore, as the result of the evolution of parthenogenesis, the developmental mechanism that forms sex-pheromone glands is repressed in parthenogenetic females.

## Conclusions

This study reveals that glandular tissues of scent plaque are differentiated from the fourth instar in sexual oviparous females, while parthenogenetic females lack the glandular cells. Furthermore, it was shown that the downstream steps of the mevalonate pathway are up-regulated in scent plaques on hind tibias of sexual females, although the upstream steps might occur somewhere in other body parts. In the aphid life cycle, oviparous sexual females only appear once a year, while parthenogenetic females occur throughout the rest of the annual life cycle [2–4]. Therefore, the adaptive switching mechanism that leads the development of sexual characters with functional metabolic pathways in response to environmental signals is suggested to have evolved, accompanying the evolution of cyclic parthenogenesis.

## Additional files

Additional file 1: **Table S1.** List of primer sequences for qRT-PCR. (PDF 23 kb)

Additional file 2: **Figure S1.** Thickness comparison of hind-tibial cuticle between adult parthenogenetic and oviparous females. Posterior parts of hind tibias were measured. Asterisks indicate significant difference (Student’s t-test, *P* < 0.01, *n* 10). **Figure S2.** Phylogenetic trees of the mevalonate-pathway genes with orthologs from other insects. **A:** *Acetoacetyl-CoA thiolase* (*AACT*). **B:** *HMG-CoA synthetase* (3-hydroxy-3-methylglutaryl-coenzyme A synthase, *HMGS*); **C:** *HMG-CoA reductase* (*HMGR*). **D:** *Mevalonate kinase* (*MevK*). **E:** *Phosphomevalonate kinase* (*MevPK*). **F:** *Mevalonate 5-diphosphate decarboxylase* (*MevPPD*). **G:** *isopentenyl pyrophosphate isomelase* (*IPPI*). **H:** *Farbesyl diphosphate synthetase* (*FPPS*). **Figure S3.** Relative expression levels of the enzyme genes in the whole body of parthenogenetic (PF) and oviparous females (OF). Vertical axes indicate relative expression levels. Asterisks indicate significant differences (Student’s t-test, *P* < 0.01). *AACT*: acetoacetyl-CoA thiolase. *HMGS*: HMG-CoA synthetase (3-hydroxy-3-methylglutaryl-coenzyme A synthase). *MevPK*: phosphomevalonate kinase. *MevPPD*: *mevalonate 5-diphosphate decarboxylase. IPPI*: *isopentenyl pyrophosphate isomelase. FPPS*: farnesyl diphosphate synthetase. (PDF 507 kb)
