# Supplementary material for: Pheromone gland development and monoterpenoid synthesis specific to oviparous females in the pea aphid
Source: Zoological Lett. 2018 May 11;4:9. doi: 10.1186/s40851-018-0092-0 (PMC5946545; doi:10.1186/s40851-018-0092-0)
Supplement: Supplementary file 1 — Table S1. List of primer sequences for qRT-PCR. (PDF 23 kb) [file 40851_2018_92_MOESM1_ESM.pdf]

Table S1

Gene	NCBI_Gene ID	Forward primer 5'−3'	Reverse primer 5'−3'
AACT1&2	100161259<br>100161636	CATTGAGACCAGCTTTT	CATTTCCGACAGTGACTAC
AACT3	100164451	AGAGCGTGATTTAGGAA	GCACCTCCATTAACATT
AACT4	100165942	ATCCTATTGGAGCATCT	GACATGCACCAGTGTTAC
AACT5	100162815	CGAATGGTGGTGTAGTC	GCACCATCATTTATTCC
HMGS1	100161670	AACGGTTTGCCGAGTCTGAT	TGTTCGGGAGTGAGGCGTAT
HMGS2	100165154	AACACCCAAGGTAAATG	CTATCTCTAAGGCCAGTAT
HMGR	100165462	CTCCTCCTACTCTCCAAAAA	AAAACGCTCAGCACGAT
MevK	100163305	CGCCTGGAAAAATAATC	CTGGTTTCCCGTATACAA
MevPK	100163413	AGGCCGTGTACGTGTTC	CGTGATGTAGTCCTTTCCA
MevPPD1	100158798	CCCATCACAGCAACCTA	CAGGCAACGAAGTTTCA
MevPPD2	100160652	GATTTACCATCCATAGC	ATACTCCTACAGGCACTT
IPPI1	100166744	ATGTCGCAGACTTGTGTTT	GGTTGACACTGGCTTGA
IPPI2	100167596	GTTTAGTGTCTTTTTGTTC	CGCTGTTGTAATAGTAGTT
FPPS	100144905	GGATTGGCGTTGGTATTGTC	GGCTTGCAGAATTTCCACAC
GAPDH	100169122	AAGTCAAGGAAGCCGCAGA	ATTCCCGCCTTAGCGTCA
