# Supplementary figures and images for: Pheromone gland development and monoterpenoid synthesis specific to oviparous females in the pea aphid
Source: Zoological Lett. 2018 May 11;4:9. doi: 10.1186/s40851-018-0092-0 (PMC5946545; doi:10.1186/s40851-018-0092-0)

Figure S1

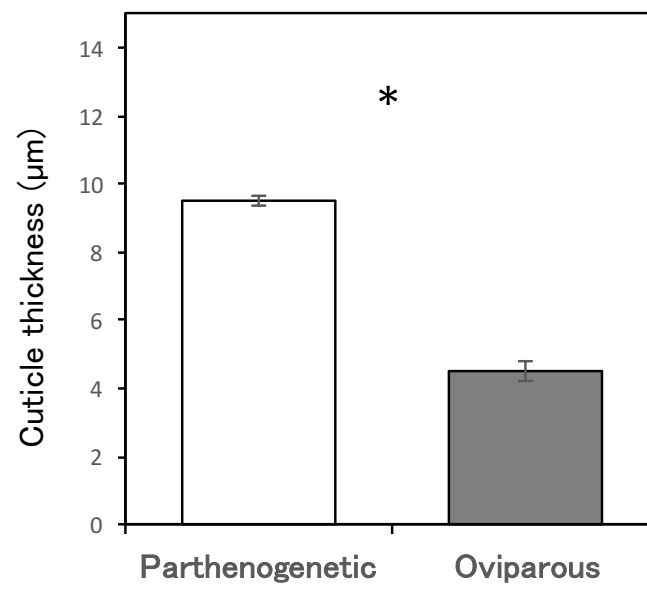

Figure S2

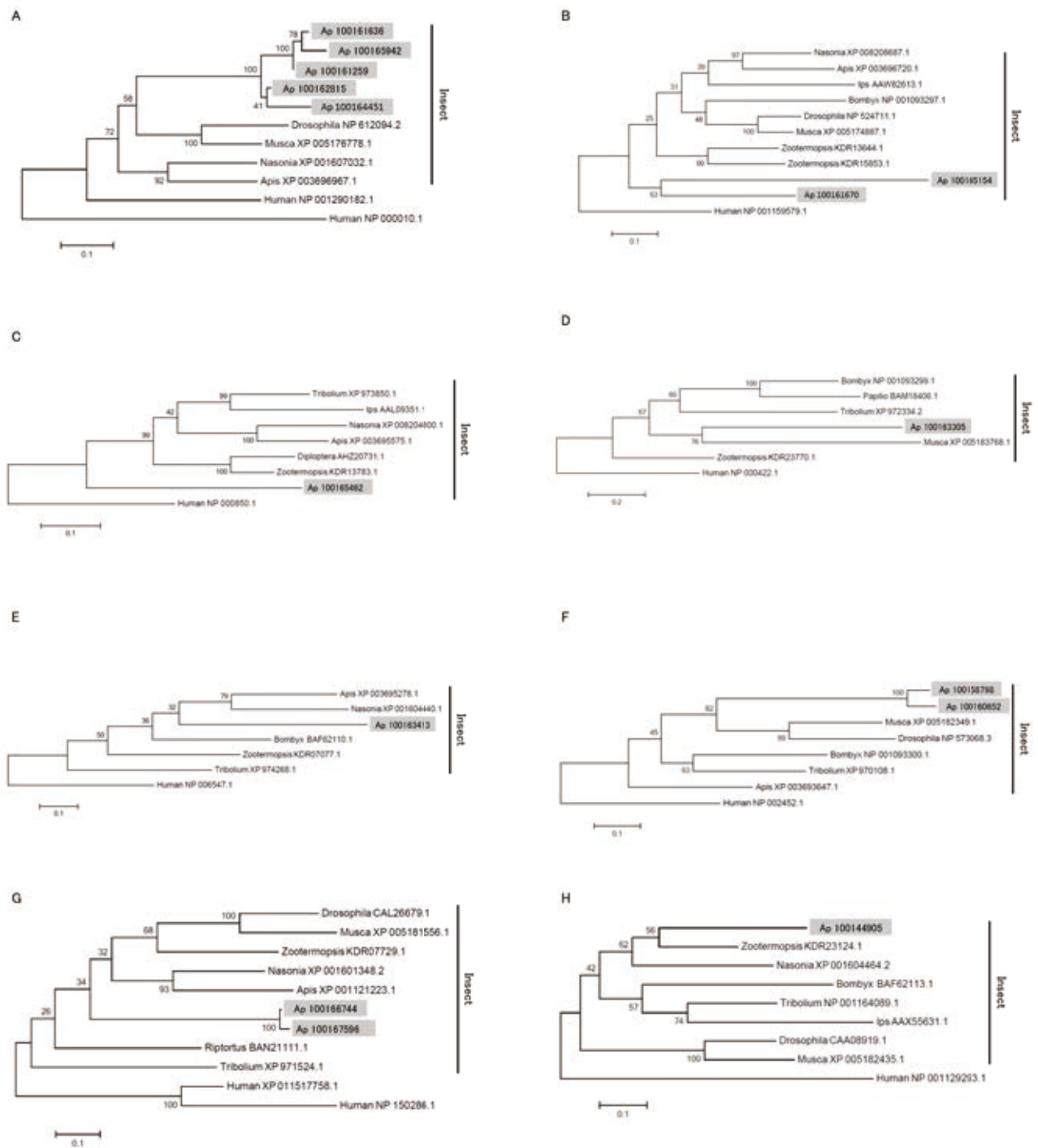

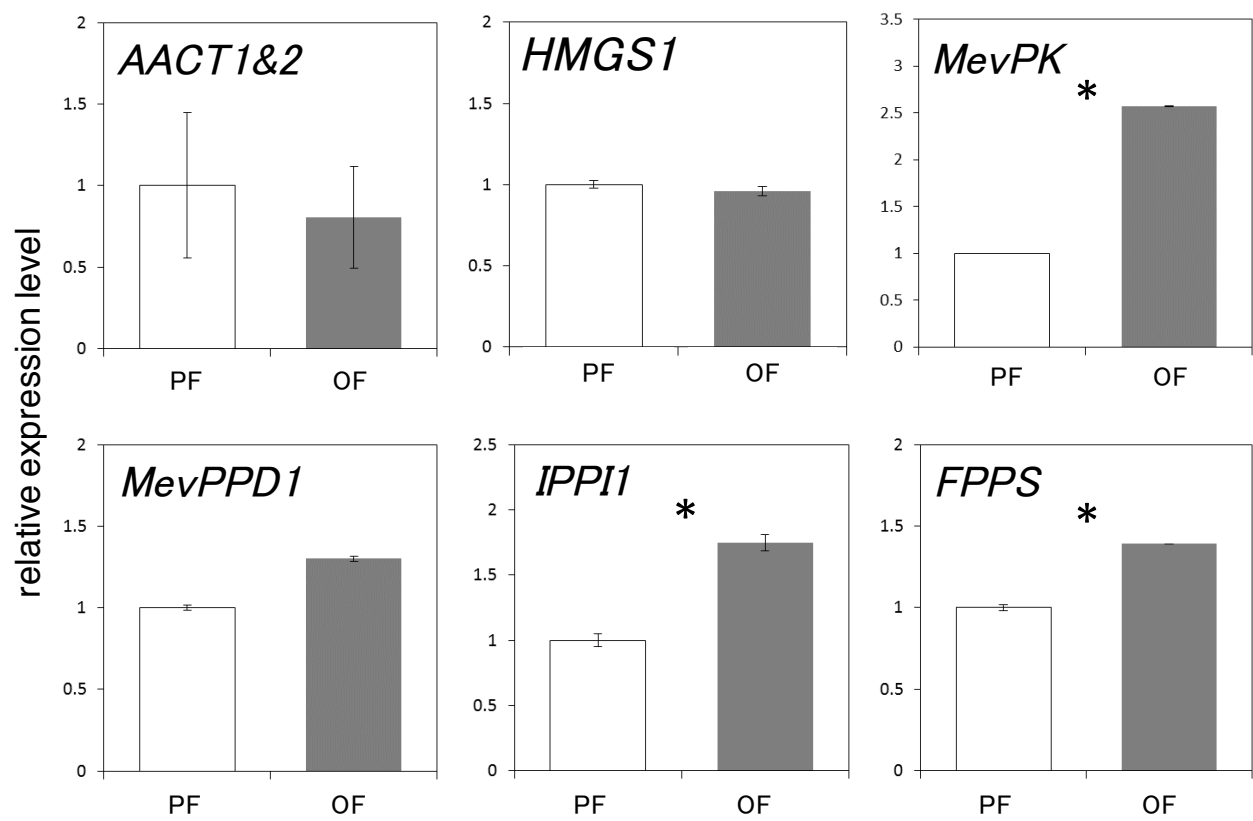

Supplement: Supplementary file 2 — Figure S1. Thickness comparison of hind-tibial cuticle between adult parthenogenetic and oviparous females. Posterior parts of hind tibias were measured. Asterisks indicate significant difference (Student’s t-test, P < 0.01, n 10). Figure S2. Phylogenetic trees of the mevalonate-pathway genes with orthologs from other insects. A: Acetoacetyl-CoA thiolase (AACT). B: HMG-CoA synthetase (3-hydroxy-3-methylglutaryl-coenzyme A synthase, HMGS); C: HMG-CoA reductase (HMGR). D: Mevalonate kinase (MevK). E: Phosphomevalonate kinase (MevPK). F: Mevalonate 5-diphosphate decarboxylase (MevPPD). G: isopentenyl pyrophosphate isomelase (IPPI). H: Farbesyl diphosphate synthetase (FPPS). Figure S3. Relative expression levels of the enzyme genes in the whole body of parthenogenetic (PF) and oviparous females (OF). Vertical axes indicate relative expression levels. Asterisks indicate significant differences (Student’s t-test, P < 0.01). AACT: acetoacetyl-CoA thiolase. HMGS: HMG-CoA synthetase (3-hydroxy-3-methylglutaryl-coenzyme A synthase). MevPK: phosphomevalonate kinase. MevPPD: mevalonate 5-diphosphate decarboxylase. IPPI: isopentenyl pyrophosphate isomelase. FPPS: farnesyl diphosphate synthetase. (PDF 507 kb) [file 40851_2018_92_MOESM2_ESM.pdf]
